# Daily supplement of sesame oil prevents postmenopausal osteoporosis via maintaining serum estrogen and aromatase levels in rats

**DOI:** 10.1038/s41598-023-50453-1

**Published:** 2024-01-03

**Authors:** Che-Chia Hsu, Po-Yen Ko, Ting-Hsien Kwan, Ming-Yie Liu, I.-Ming Jou, Chi-Wei Lin, Po-Ting Wu

**Affiliations:** 1 Department of Orthopaedics, National Cheng Kung University Hospital, College of Medicine, National Cheng Kung University, Tainan, 70428 Taiwan; 2 Department of Orthopaedics, Ditmanson Medical Foundation Chia-Yi Christian Hospital, Chiayi, 60002 Taiwan; 3 Department of Environmental and Occupational Health, College of Medicine, National Cheng Kung University, Tainan, 70101 Taiwan; 4 Department of Orthopaedics, E-Da Hospital, Kaohsiung, 82445 Taiwan; 5 School of Medicine, College of Medicine, I-Shou University, Kaohsiung, 82445 Taiwan; 6 GEG Orthopedic Clinic, Tainan, 74543 Taiwan; 7 Department of Family Medicine and Community Medicine, E-Da Hospital, I-Shou University, No.1, Yida Road, Jiao-Su Village, Yan-Chao District, Kaohsiung City, 82445 Taiwan; 8 Department of Orthopaedics, College of Medicine, National Cheng Kung University, 1 University Road, East District, Tainan City, 70101 Taiwan; 9 Department of Biomedical Engineering, National Cheng Kung University, Tainan, 70101 Taiwan; 10 Department of Biochemistry and Molecular Biology, College of Medicine, National Cheng Kung University, Tainan, 70101 Taiwan; 11 Medical Device Innovation Center, National Cheng Kung University, Tainan, 70101 Taiwan

**Keywords:** Drug discovery, Diseases

## Abstract

Estrogen deficiency is one of the main causes of postmenopausal osteoporosis in elderly women. Hormone replacement therapy has been employed to manage postmenopausal osteoporosis; however, it has raised concerns related to heart attacks and breast cancer. Sesame oil has been reported to affect sex hormone status. The aim of the present study is to evaluate the effect of sesame oil supplement on postmenopausal osteoporosis in rats. We used female Sprague Dawley rats that underwent bilaterally ovariectomy (OVX) as an experimental postmenopausal osteoporosis animal model. These rats were orally administrated sesame oil (0.25 or 0.5 mL/kg/day) for four months as the therapeutic group. We assessed bone mineral density (BMD) and the levels of osteocalcin, procollagen-I C-terminal propeptide (PICP), collagen cross-linked N-telopeptide (NTx), estradiol, and aromatase in the sera. The daily supplementation of sesame oil significantly increased BMD, serum osteocalcin levels, and trabecular areas in the OVX-treated rats. Sesame oil also elevated serum PICP levels and decreased NTx levels in these rats. Furthermore, sesame oil effectively maintained serum estradiol and aromatase levels in the OVX-induced osteoporosis rats. In conclusion, daily supplementation of sesame oil prevents postmenopausal osteoporosis by maintaining serum estrogen and aromatase levels, while also modulating the imbalance between bone formation and resorption in osteoporosis rats.

## Introduction

Postmenopausal osteoporosis is a prevalent bone disorder characterized by low bone mineral density (BMD), which significantly impacts quality of life in elderly women^1^. Osteoporosis affects over 30 million people in the United States and Europe, with numbers steadily increasing over time^2^. It often raises the risk of fragility fracture^3^. While hormone replacement therapy (HRT) has been a common treatment for postmenopausal osteoporosis, concerns such as irregular vaginal hemorrhage and breast cancer have emerged as clinical issues^4^. Therefore, safer and more efficient therapeutic interventions are needed for osteoporosis patients.

During postmenopausal osteoporosis, alternations and destruction of bone structure and homeostasis occur^5,6^. This results from an imbalance between osteoblast and osteoclast activities^7^, with estrogen deficiency after menopause typically accelerating bone turnover, where bone resorption surpasses bone formation^8^. Various mechanisms are proposed, including the role of aromatase in catalyzing testosterone into estradiol, a decrease in serum aromatase level potentially causing estradiol deficiency in females^9,10^. Additionally, ovariectomy (OVX)-induced estrogen loss can activate osteoclast, a key process in the osteoporosis pathogenesis^9^. Several well-known molecules were involved in osteoporosis. Serum alkaline phosphatase (ALP), an enzyme participating in bone mineralization, serves as the clinical biomarker for diagnosing osteoporosis^11^. Procollagen I C-terminal propeptide (PICP), a pro-peptide from newly synthesized pro-collagen, correlates with bone formation^12^. Cross-linked N-telopeptide (NTx), derived from the N-telopeptide of type I collagen, serves as a useful marker for bone resorption rates^12^.

Sesame oil, extracted from *Sesamumindicum L.*, contains fatty acids, lignans, and antioxidants and has been shown to influence sex hormone status^13^. Although a previous study demonstrated the osteoprotective effects of sesame oil, including improvements in oxidative stress, inflammation, mineral and estradiol levels in OVX rats^14^, the detailed mechanism of sesame oil’s impact on postmenopausal osteoporosis has not been extensively studied. In this study, our aim is to to explore the possible mechanisms underlying daily supplementation of sesame oil in OVX-treated rats with postmenopausal osteoporosis.

## Results

### Effects of sesame oil on histological changes in OVX-treated rats

**Figure 1:**
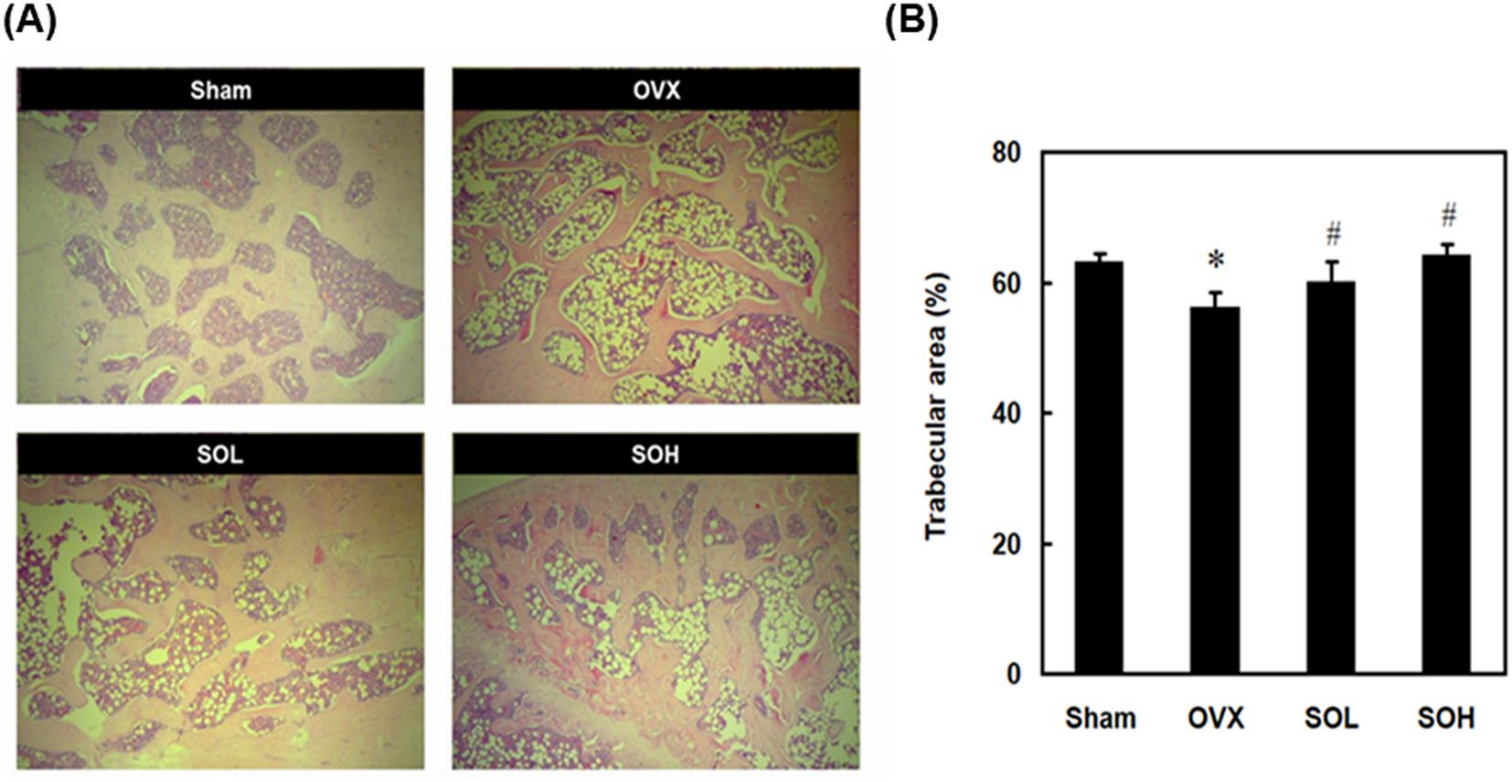
Inhibitory effects of sesame oil on OVX-induced osteoporosis. Rats were divided into four groups (n = 6). Sham group, rats received sham operation; OVX group, rats received OVX; SOL group, rats received OVX and sesame oil orally (0.25 mL/kg/day); and SOH group, rats received OVX and sesame oil orally (0.5 mL/kg/day). All animals in this study were sacrificed 4 months after sham or OVX operation. (**A**) The decalcification femurs were stained with hematoxylin and eosin. The magnifications were × 100. (**B**) The trabecular area (%) was measured with Image J software. Values were expressed as mean ± SD. *p < 0.05 compared with sham group; ^#^*p* < 0.05 compared with OVX group.

To confirm the protective effect of sesame oil on osteoporosis in OVX-treated rats, we conducted histological examinations of the trabecular areas. In the OVX group, there was a significant decrease in trabecular areas compared to the sham group. However, in both the high (SOH) and low doses (SOL) of sesame oil groups, the trabecular areas showed an increase compared to the OVX group (Fig. 1A,B).

### Effects of sesame oil on serum ALP and osteocalcin levels and total BMD in OVX-treated rats

**Figure 2:**
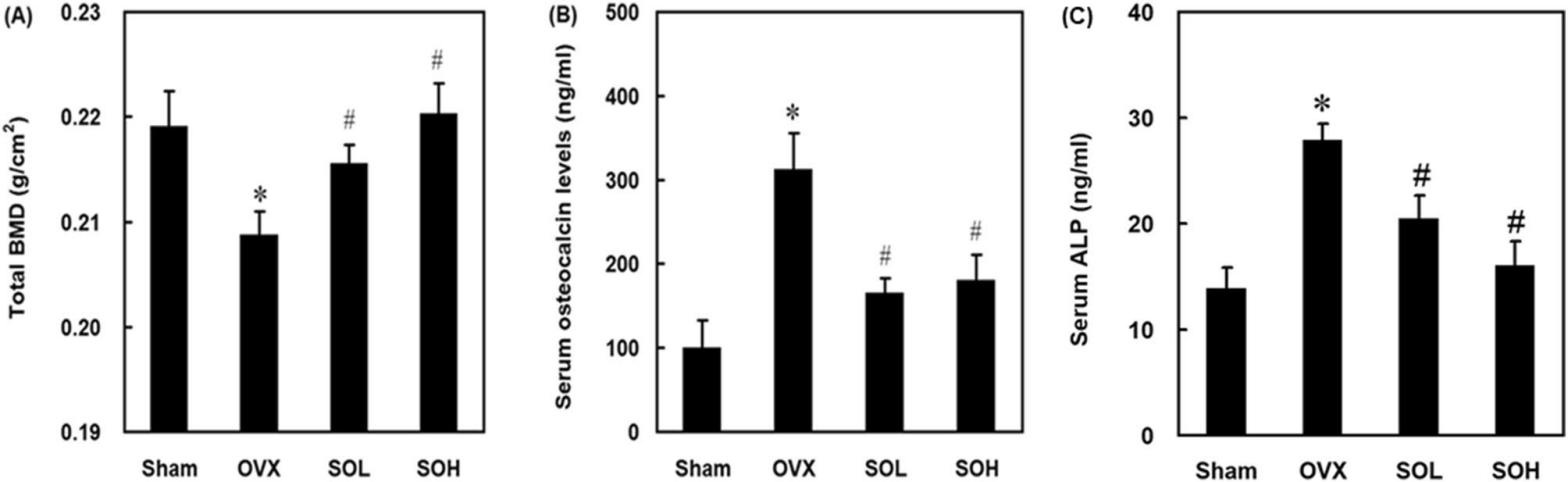
Effects of sesame oil on serum alkaline phosphatase (ALP) and osteocalcin levels and total bone mineral density (BMD) in OVX-treated rats. Rats were divided into four groups (n = 6). Sham group, rats received shame operation; OVX group, rats received OVX; SOL group, rats received OVX and sesame oil orally (0.25 mL/kg/day); and SOH group, rats received OVX and sesame oil orally (0.5 mL/kg/day). Rat (**A**) total BMD and (**B**) serum osteocalcin and (**C**) ALP levels, were determined 4 months after sham or OVX operation. Values were expressed as mean ± SD. *p < 0.05 compared with sham group; ^#^*p* < 0.05 compared with OVX group.

To investigate the impact of sesame oil on osteoporosis in OVX-treated rats, we assessed serum ALP and osteocalcin levels as well as BMD. Total BMD levels were significantly lower in the OVX group compared to the sham group. Sesame oil administration resulted in a significant increase in total BMD levels in both the SOL and SOH groups compared to the OVX group (Fig. 2A). In the OVX group, serum ALP and osteocalcin levels were significantly higher than those in the sham group. However, both SOH and SOL of sesame oil led to a significant reduction in serum osteocalcin and ALP levels compared to the OVX alone group (Fig. 2B,C).

### Effects of sesame oil on bone formation and resorption in OVX rats

**Figure 3:**
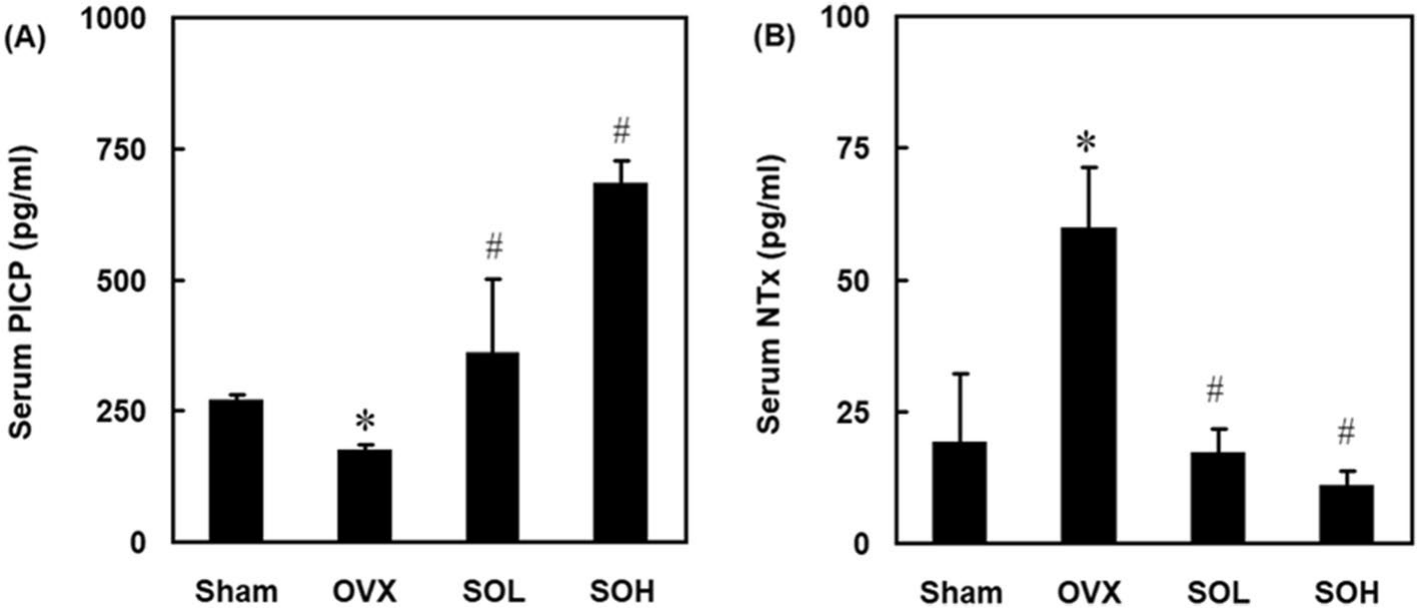
Effect of sesame oil on serum procollagen I C-terminal propeptide (PICP) and collagen cross-linked N-telopeptide (NTx) levels in OVX-treated rats. Rats were divided into four groups (n = 6). Sham group, rats received sham operation; OVX group, rats received OVX; SOL group, rats received OVX and sesame oil orally (0.25 mL/kg/day); and SOH group, rats received OVX and sesame oil orally (0.5 mL/kg/day). Serum (**A**) PICP and (**B**) NTx levels were determined 4 months after sham or OVX operation. Values were expressed as mean ± SD. *p < 0.05 compared with sham group; ^#^*p* < 0.05 compared with OVX group.

To examine the phenomena of bone formation and resorption in osteoporosis rats treated with sesame oil, serum PICP and NTx levels were determined. Serum PICP levels were significantly lower compared to the control group, while sesame oil administration significantly increased serum PICP levels compared to the OVX group (Fig. 3A). In contrast, serum NTx levels were significantly higher in the OVX group compared to the control group. However, in both the SOL and SOH groups, serum NTx levels were significantly lower compared to the OVX group (Fig. 3B).

### Effect of sesame oil on serum estradiol levels in OVX-treated rats

**Figure 4:**
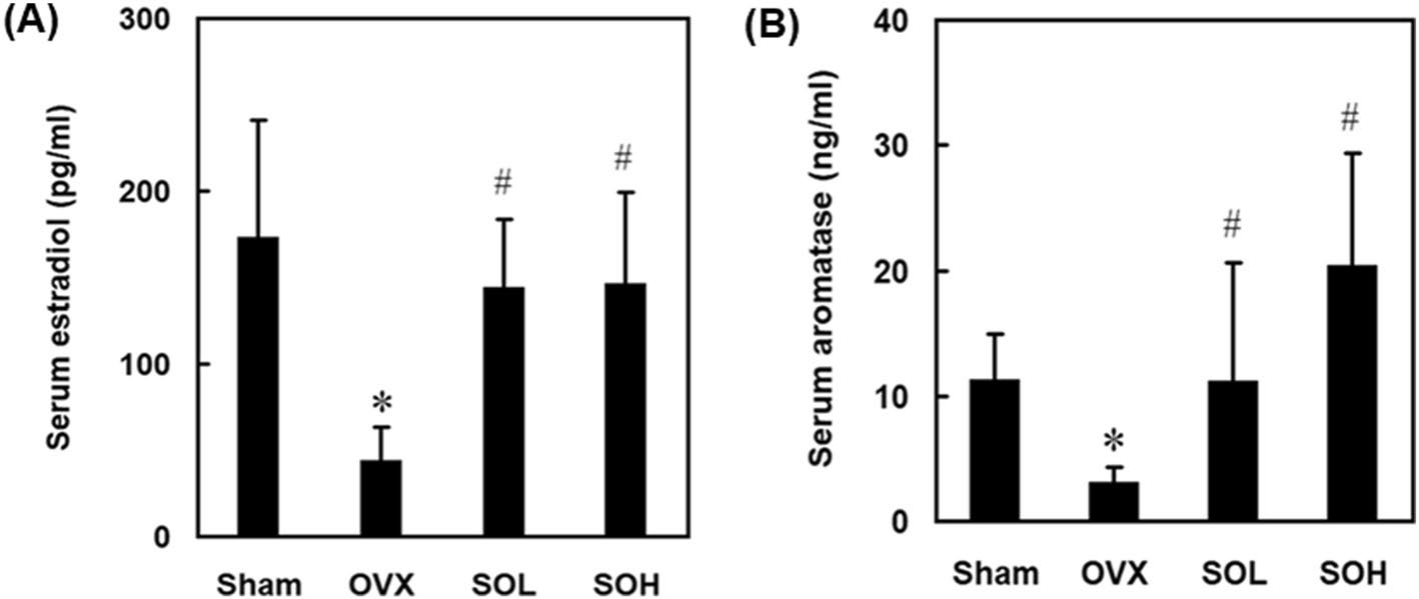
Effects of sesame oil on serum estradiol and aromatase levels in OVX-treated rats. Rats were divided into four groups (n = 6). Sham group, rats received shame operation; OVX group, rats received OVX; SOL group, rats received OVX and sesame oil orally (0.25 mL/kg/day); and SOH group, rats received OVX and sesame oil orally (0.5 mL/kg/day). Serum (**A**) estradiol and (**B**) aromatase levels were determined 4 months after sham or OVX operation. Values were expressed as mean ± SD. *p < 0.05 compared with sham group; ^#^*p* < 0.05 compared with OVX group.

To investigate the role of sexual hormone in the prevention of osteoporosis by sesame oil, serum estradiol levels were determined in the OVX-treated rats. In the OVX group, serum estradiol levels were lower compared to those in the sham group. Moreover, sesame oil administration significantly increased serum estradiol levels in both the SOL and SOH groups compared to the OVX group (Fig. 4A).

### Involvement of aromatase in sesame oil-associated estradiol alteration in OVX rats

To explore the potential mechanism responsible for the alteration of estradiol levels associated with sesame oil, OVX led to a significant decrease in serum aromatase levels compared to the sham group, whereas sesame oil administration significantly increased serum aromatase levels in both the SOL and SOH groups compared to the OVX group (Fig. 4B).

## Discussion

Postmenopausal osteoporosis is a common orthopedic disorder that significantly impacts the quality of life in women. In this study, we demonstrated that daily supplementation with sesame oil effectively prevents the development of osteoporosis in OVX rats. Sesame oil significantly increased bone formation and decreased bone resorption markers in OVX rats. Furthermore, sesame oil significantly increased serum estradiol and aromatase levels in these rats. We suggest that the increased serum aromatase levels might be one of the mechanisms responsible for maintaining estradiol levels in sesame oil-treated OVX rats.

It's important to note that while serum ALP can originate from various tissues, including the bone, liver, spleen, and placenta, research has shown that serum ALP levels are significantly higher in post-menopausal women compared to pre-menopausal women^11^. This phenomenon is also observed in osteoporosis rats^15^. In this study, daily supplementation with sesame oil was found to effectively reduce serum ALP levels while increasing total BMD and serum osteocalcin levels in rats treated with OVX. These findings suggest that sesame oil may have a beneficial effect in attenuating OVX-induced osteoporosis. Furthermore, sesame oil was found to increase serum PICP levels and decrease NTx levels. Taken together, these results suggest that sesame oil may help maintain a balance between osteoblast and osteoclast activity, which could play a significant role in the prevention of osteoporosis associated with sesame oil.

Maintaining serum estrogen levels is a crucial factor in understanding the mechanism underlying sesame oil-associated osteoprotection. Estrogen deficiency is a well-documented risk factor for osteoporosis pathogenesis^16,17^. Estrogen deficiency leads to decreased osteoblastic and increased osteoclastic activities, both of which contribute to post-menopausal osteoporosis^18^. In the present study, sesame oil was observed to increase serum estradiol levels in OVX-treated rats. One possibility is that sesame oil contains lignans, which constitute other phytoestrogenic compounds^19^. Phytoestrogens offer an alternative to estrogen replacement therapy due to their reduced side effects^20^. The most well-known phytoestrogens are isoflavones, which are abundant in soybean oil^21^. Research has been reported that isoflavones can decrease the RANKL gene expression^22^ and exhibit proapoptotic effects on osteocalsts^23^, while also increasing the OPG gene expression in osteoblasts^24^. Notably, genistein aglycone, a non-glucoside isoflavone, has been shown to decrease the RANKL/OPG ratio in OVX rats^25^ and has positive effects on BMD in osteopenic postmenopausal women^26^. As the beneficial effects of sesame seeds are primarily attributed to its lignans, especially sesamin^27^, it has also been reported that sesamin can stimulate osteoblast differentiation by increasing the OPG/RANKL ratio^28^. Furthermore, an interesting finding indicates that both soybean oil and sesame oil have anti-inflammatory, anti-oxidative stress and osteoprotective effects, while also maintaining serum estradiol levels in osteoporosis rats^14^. In addition to these effects, we propose that there may be additional mechanisms involved in attenuating OVX-induced osteoporosis through sesame oil.

The activation of the aromatase-related pathway may contribute to the maintenance of serum estrogen levels in rats treated with sesame oil after OVX. Aromatase plays a crucial role in conversing androgen to estrogen in various tissues, including the gonads, ovary, and adipocytes^9^. In our study, sesame oil effectively preserved serum estradiol and aromatase levels in OVX-treated rats. Inhibition of aromatase activity in non-pregnant female rats is a well-established animal model for polycystic ovary syndrome (PCOS). The protective effects of sesame oil on these rats are mediated by the amelioration of regulatory proteins for ER stress, lipogenesis, and steroidogenesis through the PI3K/PKA or MAPK/ERK2 signaling pathway^29^. Therefore, the associations between the PI3K/PKA or MAPK/ERK2 pathway and aromatase level may be involved in the therapeutic effects of sesame oil on female-dominant diseases. Interestingly, a study has indicated that insulin-like growth factor-I (IGF-I) stimulates the expression of estrogen receptor beta (ERβ) and aromatase through the PI3K/AKT pathway in endometriosis^30^. We propose that the mechanism underlying sesame oil-induced expression of aromatase may also work through the PI3K/AKT pathway. This mechanism could mediate the conversion of androgen to estrogen and increase the expression of ERβ, thereby enhancing the estrogen-mediated signaling pathway in osteoporosis.

Another interesting topic in osteoporosis patients is an imbalance in the gut microbiota (GM)^31,32^. The OVX mice has revealed a strong association between GM and bone mass^33^. Therefore, probiotics like *Lactobacillus* and *Bifidobacterium* have been shown to prevent bone loss induced by OVX^34–36^. Interestingly, *Lactobacillus*-fermented products, such as milk and soy skim milk, have been reported to have a beneficial effect on bone health in OVX rats and mice^37,38^. Innovative research can be expected with combinational treatments using probiotics and sesame oil, which may have syngenetic effects on osteoporosis.

This study contains limitations. First, we did not evaluate the possible underlying mechanism of sesame oil in the RANKL/RANK/OPG pathway, which is a crucial pathway involved in bone remodeling^39^ and is considered as a therapeutic target in OVX rats^40,41^. Further studies are necessary to evaluate the role of sesame oil through this signaling pathway to increase BMD. Second, we did not evaluate the baseline BMD of each rat before the index procedure, as this could influence the assessment of BMD changes within each group. However, the between-group differences after sesame oil treatment are significant. Furthermore, only evaluation of post-therapy BMD value is common in the literatures^42–44^.

**Figure 5:**
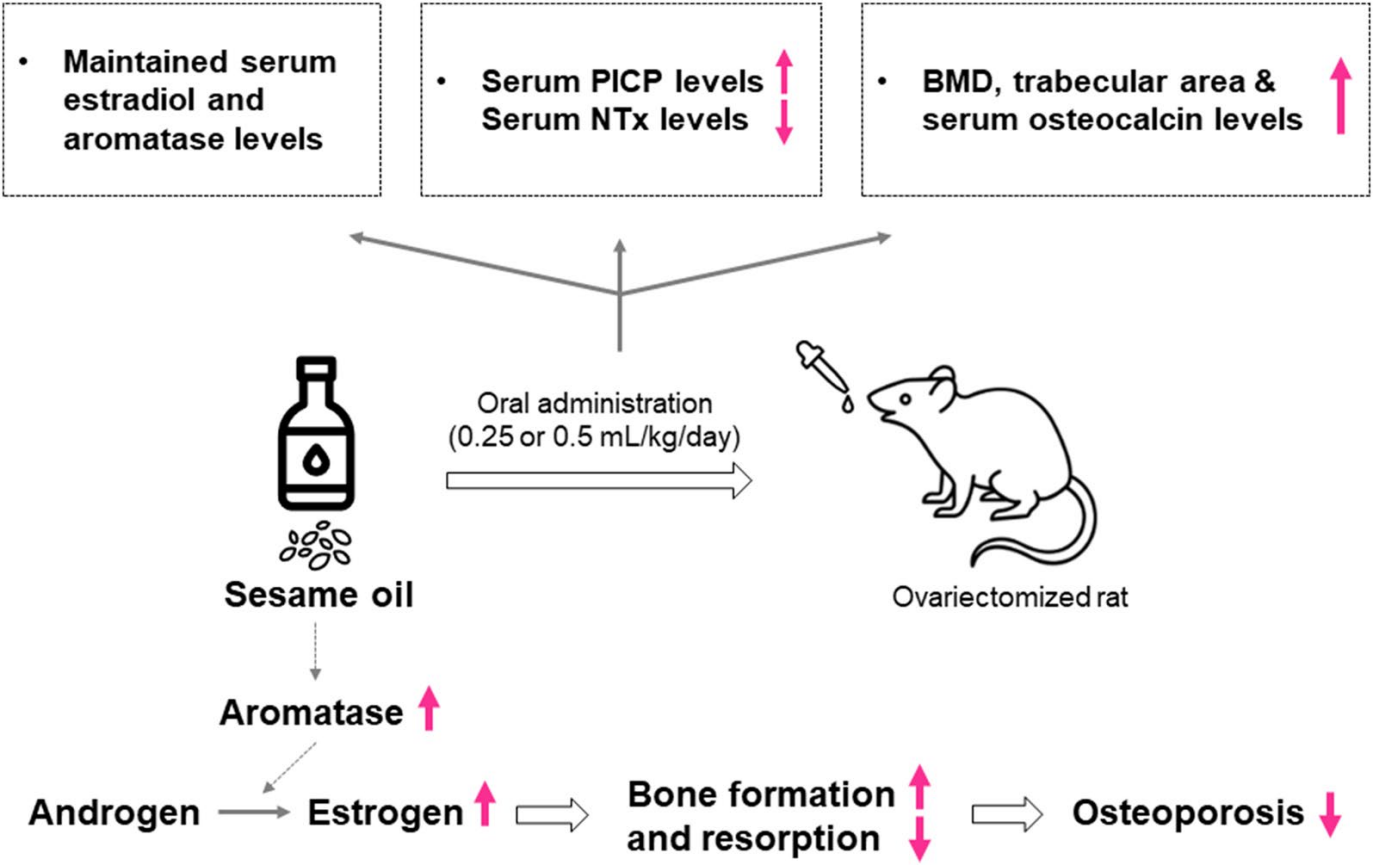
Schematic overview of the working model for sesame oil-induced osteoprotection. OVX rats were daily fed with either 0.25 mL/kg/day or 0.5 mL/kg/day of sesame oil. Increased serum aromatase levels may contribute to conversion of androgen to estrogen in sesame oil-treated OVX rats. Therefore, the increased serum PICP levels and decreased NTx represent an elevated bone formation/resorption ratio, which can lead to increased BMD in the femur, serum osteocalcin levels, and trabecular areas, thus preventing osteoporosis in OVX rats.

In conclusion, our study demonstrates that daily supplementation of sesame oil can provide osteoprotection in OVX-induced osteoporosis rats by elevating aromatase and estradiol levels (Fig. 5). These findings suggest a promising therapeutic potential for preventing postmenopausal osteoporosis in the future.

## Materials and methods

### Rat postmenopausal osteoporosis model

Twelve-week old female Sprague Dawley rats weighing between 350 and 400 g were purchased from the Laboratory Animal Center of National Cheng Kung University in Tainan, Taiwan. The rats were housed under a 12:12 light/dark cycle and provided with ad libitum access to food (5010—Laboratory Autoclavable Rodent Diet, LabDiet) and water. All animal experiments were conducted following approval by the Ethics Committee at the Laboratory Animal Center of National Cheng Kung University (IACUC Approval No. 107159). For the ovariectomy (OVX) surgery, rats were anesthetized with Zoletil-50 (Virbac, Carros, France) at a dosage of 50 mg/kg administered intraperitoneally (i.p.). The rats were then euthanized using CO_2_ inhalation at the conclusion of the experiments. To induce postmenopausal osteoporosis in rats, bilateral ovariectomy was performed by making a central incision in the lower abdomen and removing both ovaries. Rats in the sham group underwent the same surgical procedure without ovarian resection.

### Experimental groups

Twenty-four rats were divided into 4 groups: the sham group, in which rats received a sham operation; the OVX group, in which rats received OVX; the SOL group, in which OVX rats were subjected to oral administration of sesame oil (0.25 mL/kg/day), and the SOH group, in which OVX rats were subjected to oral administration of sesame oil (0.5 mL/kg/day), based on our previous studies^45^. Oral administration of sesame oil was initiated once the rats had recovered from surgery and exhibited stable vital signs. All animals in this study were sacrificed 4 months after sham or OVX operation.

### Measuring serum levels of ALP, osteocalcin, PICP, and NTx

After rat blood was collected at the end of the experiment, serum ALP levels were assessed using a blood biochemical analyzer (DRI-CHEM 3500 s; Fujifilm, Kanagawa, Japan). Serum osteocalcin levels were determined using a rat osteocalcin ELISA Kit (LifeSpan BioSciences, Seattle, WA). Serum PICP and NTx levels are the markers of bone formation and bone resorption respectively. PICP was measured by using PICP ELISA kit (Cloud-Clone Corp., Houston, TX). On the other hand, NTx was measured by using NTx ELISA kit (Cloud-Clone Corp., Houston, TX).

### Measuring BMD and bone histopathology

Rat BMD was assessed by using the Lunar Prodigy System (GE Lunar Corporation, Madison, WI, USA). The analysis was performed using enCORE 2002 (Version 6.70.021, GE, Madison, WI, USA). Rat femurs section were decalcified in 10% of EDTA-2Na for 2 weeks. The treated femurs samples were then embedded. The final blocks were sliced into sections (5 μm), and stained with hematoxylin and eosin. The trabecular area (%) was measured with Image J software as described previously^46^.

### Measuring plasma estrogen and aromatase levels

Plasma estrogen and aromatase levels were measured using immunosorbant assay kits (Cayman, Ann Arbor, MI and MyBioSource, CA, USA), followed by the manufactures’ instructions.

### Statistical analysis

Data were expressed as the means ± standard deviation (SD). The differences between groups were compared by using SPSS statistical software (SPSS Institute, Chicago, IL). In the present study, one-way ANOVA and then Tukey’s honestly significant difference post-hoc tests were used. Statistical significance was set at p < 0.05.

### Declaration of animal ethics

All animal experiments were performed in accordance with relevant guidelines and regulations by the Institutional Animal Care and Use Committee of National Cheng Kung University (approval numbers: 107159) and followed by the ARRIVE guidelines (https://arriveguidelines.org).

## Data Availability

The data that support the findings of this study are available on request from the corresponding author.
